# The Rise of Optical Coherent Tomography in Intracoronary Imaging: An Overview of Current Technology, Limitations, and Future Perspectives

**DOI:** 10.31083/RCM38123

**Published:** 2025-08-29

**Authors:** Gianluca Castaldi, Georgios Zormpas, Pascal Frederiks, Tom Adriaenssens, Johan Bennett

**Affiliations:** ^1^Department of Cardiovascular Medicine, University Hospital Leuven, 3000 Leuven, Belgium; ^2^Second Department of Cardiology, Aristotle University of Thessaloniki, Hippokration General Hospital, 546 42 Thessaloniki, Greece; ^3^Department of Cardiovascular Sciences, Katholieke Universiteit Leuven, 3000 Leuven, Belgium

**Keywords:** percutaneous coronary interventions, intravascular imaging, optical coherence tomography

## Abstract

Intravascular optical coherence tomography (OCT) has represented a revolutionary invasive imaging method, offering *in vivo* high-resolution cross-sectional views of human coronary arteries, thereby promoting a significant evolution in the understanding of vascular biology in both acute and chronic coronary pathologies. Since the development of OCT in the early 1990s, this technique has provided detailed insights into vascular biology, enabling a more thorough assessment of coronary artery disease (CAD) and the impact of percutaneous coronary intervention (PCI). Moreover, a series of recent clinical trials has consistently demonstrated the clinical benefits of intravascular imaging (IVI) and OCT-guided PCI, showing improved outcomes compared to angiography-guided procedures, particularly in cases of complex coronary pathology. Nonetheless, despite the advantages of OCT, several limitations remain, including limited penetration depth and the necessity for additional contrast agent administration, which may potentially constrain the widespread adoption of OCT. Moreover, economic and logistical challenges remain, including heterogeneous levels of training among interventional cardiologists, which leads to the underutilization of OCT in the Western world. Meanwhile, emerging technologies and the integration of machine learning and artificial intelligence-based algorithms are set to enhance diagnostic accuracy in daily practice. Future research is necessary to address existing limitations and investigate next-generation devices, further advancing the field of interventional cardiology toward optimal imaging-guided PCI and improved outcomes.

## 1. Introduction

Coronary artery disease (CAD) represents one of the leading causes of morbidity and mortality in the Western world, posing a significant burden on patients and healthcare systems [1, 2]. In this context, percutaneous coronary intervention (PCI) represents the cornerstone treatment of obstructive CAD. Since the first PCI in 1977, there has been a constant drive to develop the technique further, integrate novel therapeutic tools, and improve short- and long-term clinical outcomes [3]. Consequently, intravascular imaging (IVI) technologies, such as intravascular ultrasound (IVUS) and optical coherence tomography (OCT), have been developed to enhance the understanding of the vessel wall anatomy beyond the limitations of coronary angiography [4]. IVI has subsequently emerged as a revolutionary advancement, comprising a near-histological resolution imaging technique that provides detailed cross-sectional images of the coronary artery wall and overcomes the limitations of traditional coronary angiography [5].

Nonetheless, the use of IVI is not equally widespread worldwide. According to the latest data available from national- and industry-based registries, IVI is used in ∼85% of PCI cases in Japan compared to ∼25% in UK (the highest in the western countries), 15% in the USA and 10–15% in most European countries [6, 7, 8, 9], with great variability among centers in each country. The issue of the low IVI uptake is multifaceted. Generally, the reimbursement framework is one of the major barriers to the adoption of new technologies. In Japan, the approval of new medical devices is based on non-inferiority criteria; in most other countries, reimbursement policies often require extensive cost-effectiveness and clinical utility data before approval [10]. This, for example, led to the early adoption of IVUS in Japan, with reimbursement beginning in 1994, which paved the way for the widespread national application of the technology. Administrators may also be reluctant to invest in IVI technologies due to the associated costs, particularly if the perceived return on investment is unclear. Despite the differences in reimbursement protocols between countries and between healthcare models, several studies have already shown a favorable cost–benefit impact of IVI [11, 12, 13]. However, while reimbursement policies are key limits for a broader uptake of these tools, other relevant barriers continue to impact IVI. Adequate exposure to IVI during the training period is critical, as the additional information derived from intracoronary imaging can be overwhelming for operators not accustomed to the technique. Furthermore, discerning what matters most and reacting accordingly can also be challenging. Moreover, operators may be convinced that their angiography-guided procedures are already optimal, while the same operator rarely witnesses longer-term PCI-related events. IVI challenges these beliefs and may create cognitive dissonance [14]: operators may try to avoid situations and information that would likely increase such conflicts with their convictions. Important steps can be taken to overcome these obstacles further and support a wider adoption of IVI: (1) the development of enhanced education and training models to improve imaging interpretation skills; (2) dissemination of the existing health economic data and create new evidence to implement current clinical practice guidelines recommendations and dispel the assumption of increased cost as a barrier to utilization; (3) fostering the engagement and training of the whole team in the catheterization laboratory, including nurses and technicians, to optimize the workflow and make the use of IVI even more efficient [6].

Despite these barriers, the use of IVI and OCT is crucial in healthcare to ensure the best outcomes possible for patients. The current review aims to explore the evolution of OCT technology and evaluate its current applications and remaining limitations. Furthermore, the emergence of next-generation devices that can further expand the utility of this technology will be discussed.

## 2. The Evolution of OCT

Intravascular OCT is an imaging modality that uses near-infrared light to provide high-definition, cross-sectional, and three-dimensional (3D) images of the coronary arteries. This method was first conceived in 1991 by Professor James Fujimoto at Massachusetts Institute of Technology (MIT), while the technology was further developed and integrated into clinical practice in 1998 by a team that included MIT scientists Brett Bouma and Guillermo Tearney, along with cardiologist Ik-Kyung Jang, at Massachusetts General Hospital [15]. The first clinically available devices utilized time-domain detection (TD-OCT) for image acquisition, resulting in a slow image acquisition rate (maximum pullback speed of 2 mm/s) and requiring proximal vessel occlusion and distal saline infusion through a dedicated balloon. These important technological drawbacks were overcome in 2006 with the introduction of frequency-domain OCT (FD-OCT), which introduced a more user-friendly device with faster image acquisition (20 mm/s) and without the need for complete blood flow occlusion to the field. Within the last few decades, OCT has facilitated a more comprehensive understanding of vascular biology thanks to the high spatial resolution (10–20 µm), which has provided detailed information on the pathophysiology of atherosclerosis and the underlying physiopathological mechanisms of CAD, such as plaque erosion, neo-atherosclerosis, and stent thrombosis [16]. Additionally, OCT is a valuable tool for guiding PCI and optimizing outcomes, especially in more complex scenarios, such as bifurcations and calcified lesions. OCT allows more effective visualization and characterization of calcified lesions, providing valuable information on which the technique can be more appropriate to use for plaque modification (e.g., rotational atherectomy, intravascular lithotripsy, orbital atherectomy, or cutting and scoring balloons), and achieving improved outcomes in patients with complex coronary artery lesions [17].

A large series of registries, randomized clinical trials (RCTs), and meta-analyses support the use of OCT in the catheterization laboratory. Therefore, every interventional cardiologist needs to be well-acquainted with this technology.

## 3. IVUS vs. OCT

**Fig. 1.**
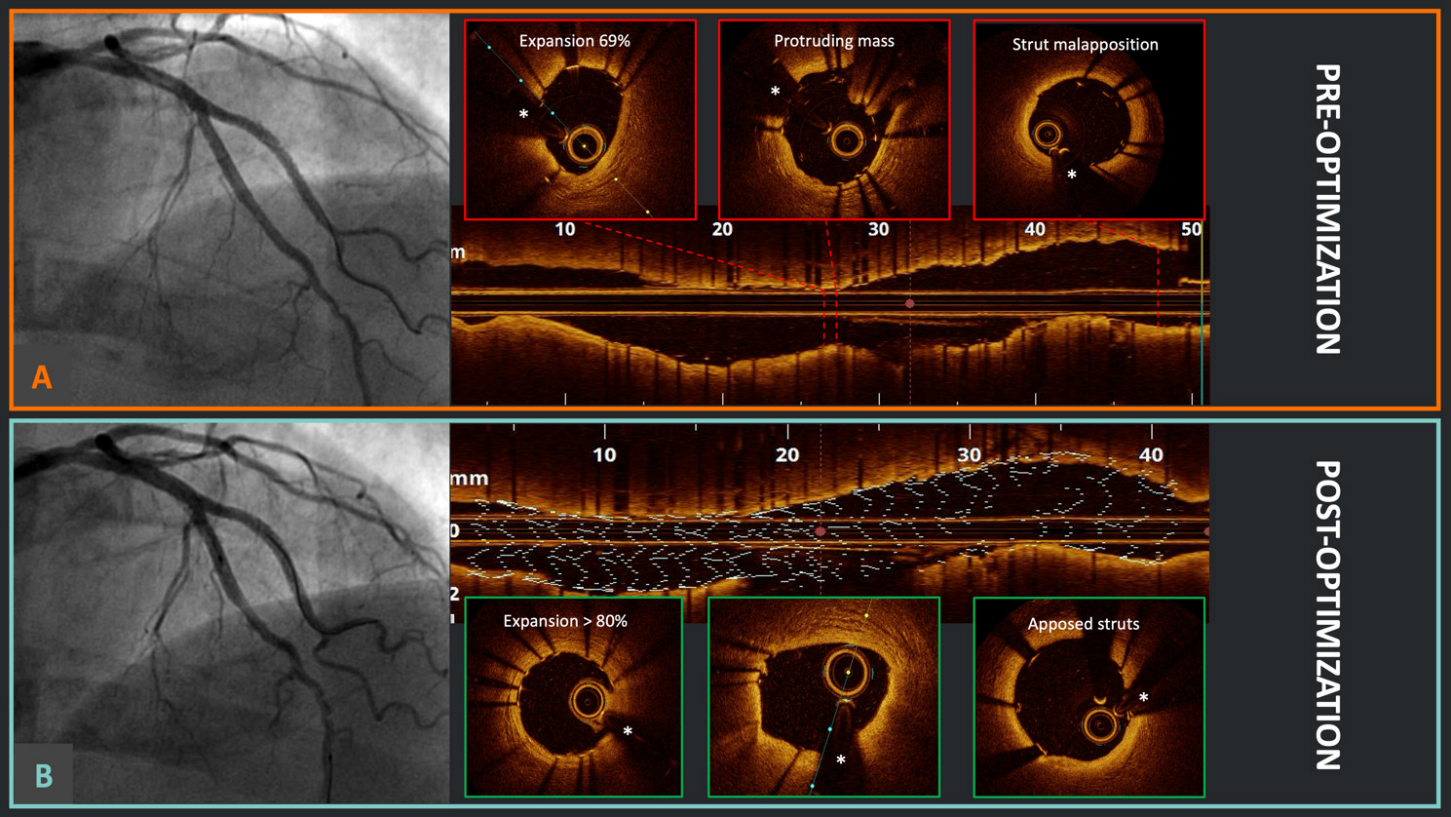
**Optical coherence tomography (OCT)-guided percutaneous coronary intervention (PCI) optimization**. (A) Good angiographic result after double kissing crush technique for a left anterior descending artery and diagonal branch bifurcation in the Optical Coherence Tomography Optimized Bifurcation Event Reduction (OCTOBER) study; meanwhile, the OCT pullback identified the presence of stent underexpansion, a protruding mass (likely calcium), and relevant proximal strut malapposition. (B) After OCT-guided optimization, there was no significant angiographic difference; however, at OCT, it is evident that the stent underexpansion has been successfully corrected, with no protruding masses and appropriate strut apposition proximally. *Wire artifact.

In the spectrum of IVI, two essential modalities are available: IVUS and OCT, each with distinct principles and technical specifications. IVUS is based on the propagation and reflection of high-frequency sound waves within coronary vessels to generate cross-sectional images. The first phased-array IVUS catheters were introduced in the clinical field in the early 1990s, with low-resolution imaging (20–40 MHz, 100–200 µm axial resolution). More recently, high-frequency (up to 65 MHz) and dual-frequency catheters (35/65 MHz), developed using asymmetric electrodes for improved beam profiles, have been introduced, allowing higher-resolution quality imaging (22–40 µm axial resolution) while maintaining deep tissue penetration [15]. Conversely, OCT operates on the principles of low-coherence interferometry, employing near-infrared light-emitting catheters that typically work with wavelengths around 1300 nm. These beams of light diverge into two arms: one directed toward the coronary tissue under examination (the sample arm) and another toward a reference mirror (the reference arm). Light reflected from both the sample and reference arms recombines, creating an interference pattern. OCT measures the interference pattern to construct detailed cross-sectional images. This pattern is highly sensitive to variations in the path length of the reflected light, allowing OCT to achieve an exceptional resolution [16, 17]. However, due to their technical specificities and differences in clinical practice, their application does not completely overlap [18]. IVUS offers a superior penetration depth, especially relevant in large vessels (e.g., the left main), and is instrumental in characterizing coronary wall remodeling secondary to CAD progression (i.e., positive vs. negative remodeling). Since no additional administration of contrast is necessary, IVUS can be crucial for assessing and treating spontaneous coronary artery dissections in cases of uncertain angiographic diagnosis [19] or for navigating and wiring the intra- and extraluminal spaces in cases of chronic total occlusions [20, 21]. Finally, IVUS can be an exceptional tool to achieve optimal results in patients with severe renal dysfunction and complex lesions that require intervention [22]. OCT stands out with its exceptional resolution, enabling the precise assessment of microstructures within the coronary vessel wall, which is crucial for evaluating plaque vulnerability, macrophage infiltration, and detecting the most subtle intimal disruptions [23]. Moreover, in cases of a high calcium burden, OCT allows a more precise analysis of plaque distribution and differentiation [24]. Finally, the detailed visualization of coronary stents, whether immediately after implantation or at longer-term follow-up, precise stent optimization during PCI, and the assessment of strut coverage and stent failure at follow-up are specific applications where OCT can be of great benefit [25, 26]. An example of OCT-guided stent optimization is presented in Fig. 1. A technical comparison between current-generation IVUS and OCT systems, along with respective potential advantages in different clinical settings, is shown in Table 1.

**Table 1.** **Comparison between current-generation IVUS and OCT devices**.

		IVUS	OCT
Technical comparison			
	Energy source	Ultrasound	Near-infrared light
	Frequency (MHz) - wavelength (nm)	HD 40–65 MHz	1310 nm
		non-HD 20 MHz	
	Spatial axial resolution	HD 22–40 µm	10–20 µm
		Non-HD 100–200 µm	
	Spatial lateral resolution	50–200 µm	40 µm
	Soft tissue penetration	4–8 mm	1–3 mm
	Pullback speed	0.5–10 mm/s	18–36 mm/s
	Pullback length	Up to 150 mm	Up to 75 mm
	Elimination of blood	No	Yes
Clinical utility			
	Lipid core	++	+++
	Fibrous cap	+	+++
	Remodeling	+++	–
	Calcium assessment	++	+++
	Thrombus	+	++
	Macrophage	–	++
	Plaque erosion	+	+++
	Intermediate lesion morphological assessment	++	++
	Post-PCI assessment	+++	+++

+: assessment possible, good indication; ++: assessment clinically relevant, very good indication; +++: assessment critical, optimal indication; –: assessment not possible. OCT, Optical coherence tomography; IVUS, intravascular ultrasound; HD, high definition; PCI, percutaneous coronary intervention.

Based on the above comparisons between OCT and IVUS and preliminary findings, the following section will explore outcome studies on OCT-guided PCI in greater detail.

## 4. Clinical Evidence for OCT-Guided PCI

Numerous observational studies have indicated that PCI guided by OCT yields superior clinical outcomes when compared to angiography-guided PCI [27, 28, 29]. Recently, the advantages of OCT-guided PCI have been explored in several significant RCTs, with a particular emphasis on complex coronary artery lesions [30, 31, 32, 33]. Additionally, meta-analyses of some of these trials have been published [34, 35, 36, 37]. An overview of these landmark studies and meta-analyses is provided in Table 2 (Ref. [30, 31, 32, 33, 34, 35, 36, 37, 38, 39, 40]).

**Table 2.** **Randomized controlled trials and meta-analyses of OCT-guided PCI**.

		Study population and intervention	Primary endpoint(s)
Randomized controlled trial (year)			
	ILUMIEN III (2017) [38]	450 pts randomized to OCT-guided (n = 158) vs. IVUS-guided (n = 146) vs. angiography-guided (n = 146) PCI	Post-PCI MSA in OCT
	OPINION (2021) [39]	829 pts randomized to OCT-guided (n = 414) vs. IVUS-guided (n = 415) PCI	TVF (cardiac death, target-TV-MI, and ischemia-driven TVR) at 1 year
	RENOVATE-COMPLEXPCI (2023) [30]	1639 pts with complex coronary artery lesions randomized to IVI-guided (n = 1092: 800 IVUS, 278 OCT) vs. angiography-guided (n = 547) PCI	TVF (cardiac death, TV-MI or clinically driven TVR) at a median of 2.1 years
	ILUMIEN IV (2023) [32]	2487 pts with complex coronary artery lesions randomized to OCT-guided (n = 1233) vs. angiography-guided (n = 1254) PCI	(1) Post-PCI MSA in OCT
			(2) TVF (cardiac death, TV-MI, or ischemia-driven TVR) at 2 years
	OCTOBER (2023) [31]	1201 pts with complex true bifurcation lesions randomized to OCT-guided (n = 600) vs. angiography-guided (n = 601) PCI	MACEs (cardiac death, TL-MI, or ischemia-driven TLR) at 2 years
	OCTIVUS (2023) [40]	2008 pts randomized to OCT-guided (n = 1005) vs. IVUS-guided (n = 1003) PCI	TVF (cardiac death, TV-MI, or ischemia driven TVR) at 1 year
	OCCUPI (2024) [33]	1604 with complex coronary artery lesions randomized to OCT-guided (n = 803) vs. angiography-guided (n = 801) PCI	MACEs (cardiac death, MI, ST, and ischemia-driven TVR) at 1 year
Meta-analyses			
	Khan *et al*. (2023) [34]	20 RCTs	Cardiac death, MI, ST, TVR, or TLR
		11,698 pts	
		IVI-guided (IVUS or OCT) vs. angiography-guided PCI	
	Kuno *et al*. (2023) [35]	32 RCTs	MACEs (cardiac death, MI, and TLR)
		22,684 pts	
		IVI-guided or functionally-guided PCI vs. angiography-guided PCI	
	Giacoppo *et al*. (2024) [36]	24 RCTs	TLR and MI
		15,489 pts	
		IVUS vs. angiography, 7189 pts (46.4%); OCT vs. angiography, 4976 pts (32.1%); OCT vs. IVUS, 3324 pts (21.4%)	
	Stone *et al*. (2024) [37]	22 RCTs	TLF (cardiac death, TV-MI, or TLR)
		15,964 pts	
		IVI-guided (IVUS or OCT) vs. angiography-guided PCI; weighted mean follow-up duration of 24.7 months	

OCT, optical coherence tomography; IVUS, intravascular ultrasound; IVI, intravascular imaging; MACEs, major adverse cardiovascular events; MI, myocardial infarction; MSA, minimum stent area; PCI, percutaneous coronary intervention; RCT, randomized controlled trial; TLF, target lesion failure; TLR, target lesion revascularization; TV-MI, target vessel-related myocardial infarction; TVF, target vessel failure; TVR, target vessel revascularization; ST, stent thrombosis.

The Randomized Controlled Trial of Intravascular Imaging Guidance versus Angiography-Guidance on Clinical Outcomes after Complex Percutaneous Coronary Intervention (RENOVATE-COMPLEX-PCI) study, which compared intravascular imaging guidance to angiography guidance in complex PCI, found that imaging-guided PCI (IVUS in 74% of cases and OCT in 26%) resulted in a reduced risk of the primary composite endpoints, including cardiac death, target vessel-related myocardial infarction (TV-MI), and clinically driven target vessel revascularization (TVR) [30]. The results of the primary endpoint analyses were similar in the patients who underwent OCT or IVUS (53% reduction of primary events with OCT and 44% reduction with IVUS compared with angiography alone).

In the Optical Coherence Tomography Optimized Bifurcation Event Reduction (OCTOBER) study, 1201 patients with complex coronary artery bifurcation lesions were randomized to OCT-guided PCI or angiography-guided PCI [31]. At a median follow-up of 2 years, the incidence of the primary composite endpoint of target lesion failure (TLF), defined as death from cardiac cause, target lesion MI, or ischemia-driven target lesion revascularization (TLR), was significantly lower in the OCT-guided group than in the angiography-guided group (10.1% and 14.1%, respectively; *p* = 0.035). In the ILUMIEN IV: OPTIMAL PCI trial, 2487 patients with either medically-managed diabetes or complex coronary artery lesions were randomly allocated to undergo OCT-guided or angiography-guided PCI [32]. In the angiography group, a final blinded OCT procedure was performed. One of the primary efficacy outcomes showed that OCT guidance led to a larger minimum stent area (MSA) post-PCI compared to angiography guidance (5.72 ± 2.04 mm^2^ vs. 5.36 ± 1.87 mm^2^, respectively). Surprisingly, this increase in MSA did not correspond to a significant decrease in the primary clinical endpoint of TVF at the 2-year follow-up (7.4% vs. 8.2%, respectively; *p* = 0.45). However, the occurrence of stent thrombosis within two years was notably lower in the OCT group compared to the angiography group, at 0.5% and 1.4%, respectively (*p* = 0.02). Finally, the OCCUPI trial was an investigator-initiated, multicenter, randomized, open-label, superiority trial conducted in South Korea [33]. A total of 1604 patients with complex coronary lesions were randomly assigned to either OCT-guided PCI (803 patients) or angiography-guided PCI (801 patients). The primary endpoint, major adverse cardiovascular events (MACEs) (composite of cardiac death, myocardial infarction, stent thrombosis, or ischemia-driven TVR) at the 1-year follow-up occurred in 5% of the OCT-guided group compared to 7% in the angiography-guided group, indicating a statistically significant reduction in MACEs with OCT guidance (HR, 0.62; *p* = 0.023). This reduction was largely driven by significant decreases in ischemia-driven TVR (HR, 0.36; *p* = 0.0022) and spontaneous myocardial infarction (HR, 0.36; *p* = 0.022) in the OCT group.

Previously, several studies have compared the clinical effectiveness of OCT versus IVUS-guided PCI, showing no significant difference in terms of MSA, stent expansion, or TVF at 1 year; however, these studies were underpowered in low-risk patients and simpler lesions [38, 39]. More recently, the Optical Coherence Tomography Versus Intravascular Ultrasound Guided Percutaneous Coronary Intervention (OCTIVUS) study conducted a direct comparison between OCT-guided and IVUS-guided PCI in patients with a broad range of coronary artery lesions [40]. The main findings indicated that OCT-guided PCI was not inferior to IVUS-guided PCI concerning the primary composite endpoint of TVF at one year, with rates of 2.5% and 3.1%, respectively (*p* < 0.001 for non-inferiority). Although the OCT-guided group used a larger amount of contrast dye, the occurrence of contrast-induced nephropathy was comparable between the two groups (1.4% in the OCT group vs. 1.5% in the IVUS group; *p* = 0.85). Meanwhile, the rate of major procedural adverse events was lower in the OCT group compared to the IVUS group (2.2% vs. 3.7%; *p* = 0.047), despite no events being directly linked to the imaging procedures in either group.

Finally, recently published meta-analyses have consistently shown that imaging-guided PCI, with either OCT or IVUS, is associated with reduced risks of MACEs or mortality compared with angiography-guided PCI, while no significant differences in efficacy or safety between IVUS- and OCT-guided PCI were evident [34, 35, 36, 37].

These findings add to the growing body of evidence that supports the use of intravascular imaging in optimizing stent deployment and procedural outcomes, and have resulted in a Class 1A recommendation in the European Society of Cardiology (ESC) 2024 guidelines for treating chronic coronary syndromes of complex coronary diseases, such as left main disease, bifurcations, and long lesions [41]. The aforementioned landmark studies also confirmed that OCT-guided PCI is a safe and effective approach that significantly reduces MACEs compared to angiography-guided PCI, particularly through a reduction in cardiac death, myocardial infarction, repeat revascularization, and stent thrombosis. However, these trials also highlight the challenges associated with achieving consistent procedural optimization and underscore the need for further research to improve the understanding of the long-term benefits of OCT. As OCT becomes more integrated into clinical practice, its role in improving outcomes for patients with complex coronary lesions is likely to continue growing, supported by ongoing advancements in technology and operator training.

## 5. Specific Clinical Indications for Imaging With OCT

### 5.1 Evaluation of Intermediate Coronary Artery Stenosis

Intermediate coronary artery stenosis, defined as visual angiographic stenosis severity between 30% and 70%, is present in up to 25% of patients undergoing coronary angiography [42]. Although international guidelines recommend physiological pressure-based assessment of these lesions utilizing fractional flow reserve (FFR) or other indices, specific clinical scenarios and lesion subsets exist where the use of such indices may not be reliable. In the FORZA trial, the authors compared FFR and OCT for evaluating intermediate coronary artery stenoses [43]. The results of the study demonstrated similar events in the two patient groups (FFR vs. OCT) in terms of MACEs within a 5-year follow-up period. At the 1-month follow-up, FFR guidance for intermediate coronary artery lesions showed a higher frequency of medical therapy use compared to OCT guidance. However, at the 13-month follow-up, OCT guidance resulted in lower rates of MACEs and significant angina compared to FFR guidance. Frequently, left main coronary artery (LMCA) lesions are associated with downstream and/or multivessel disease. The presence of downstream disease and/or multivessel disease can potentially compromise the accuracy of FFR in assessing the true functional significance of the LMCA stenoses. Recent studies using FD-OCT have investigated the technical feasibility of OCT for assessing left main disease, showing that this technology can accurately evaluate angiographically visualized atherosclerotic plaques of the main stem and the ostia of its daughter branches [44, 45].

### 5.2 Evaluation of Stent Failure

Stent failure, either in-stent restenosis (ISR) or stent thrombosis (ST), remains one of the major challenges in contemporary interventional cardiology. Approximately 5% of all PCIs are performed to treat ISR lesions [46]. Thus, the use of intravascular imaging, especially OCT, is extremely helpful for managing ISR, as it plays a crucial role in identifying the mechanism through which stent failure occurred and guiding subsequent treatment strategies [47]. In particular, OCT can provide detailed information on distinct mechanisms of ISR, such as stent underexpansion, neointimal hyperplasia, and neoatherosclerosis. Moreover, compared to IVUS, OCT has a superior ability to define plaque morphology, expansion of the original stent, and calcium burden outside the stent, as reported in recent key analyses in the OCTIVUS trial [48]. Despite the low overall rates of early and late ST with modern generation drug eluting stent (DES) and the contemporary antithrombotic therapies (from ~3.0% with first generation DES up to the 3-year follow-up to 1.5% with second generation) [49], the large number of patients treated daily worldwide makes this condition a significant health issue, which often results in myocardial infarction. In the vast majority of ST cases, at least one underlying morphological abnormality can be identified using OCT, including major underexpansion, malapposed or uncovered struts, neoatherosclerosis, and edge-related disease progression [50, 51]. In this setting, identifying the mechanism of ST is crucial for tailoring the most appropriate therapy. Therefore, recent clinical guidelines have proposed a Class IIa recommendation (level of evidence C) for the use of OCT in determining the mechanism of stent failure [52].

### 5.3 Assessment of the Mechanism in Acute Coronary Syndrome

**Fig. 2.**
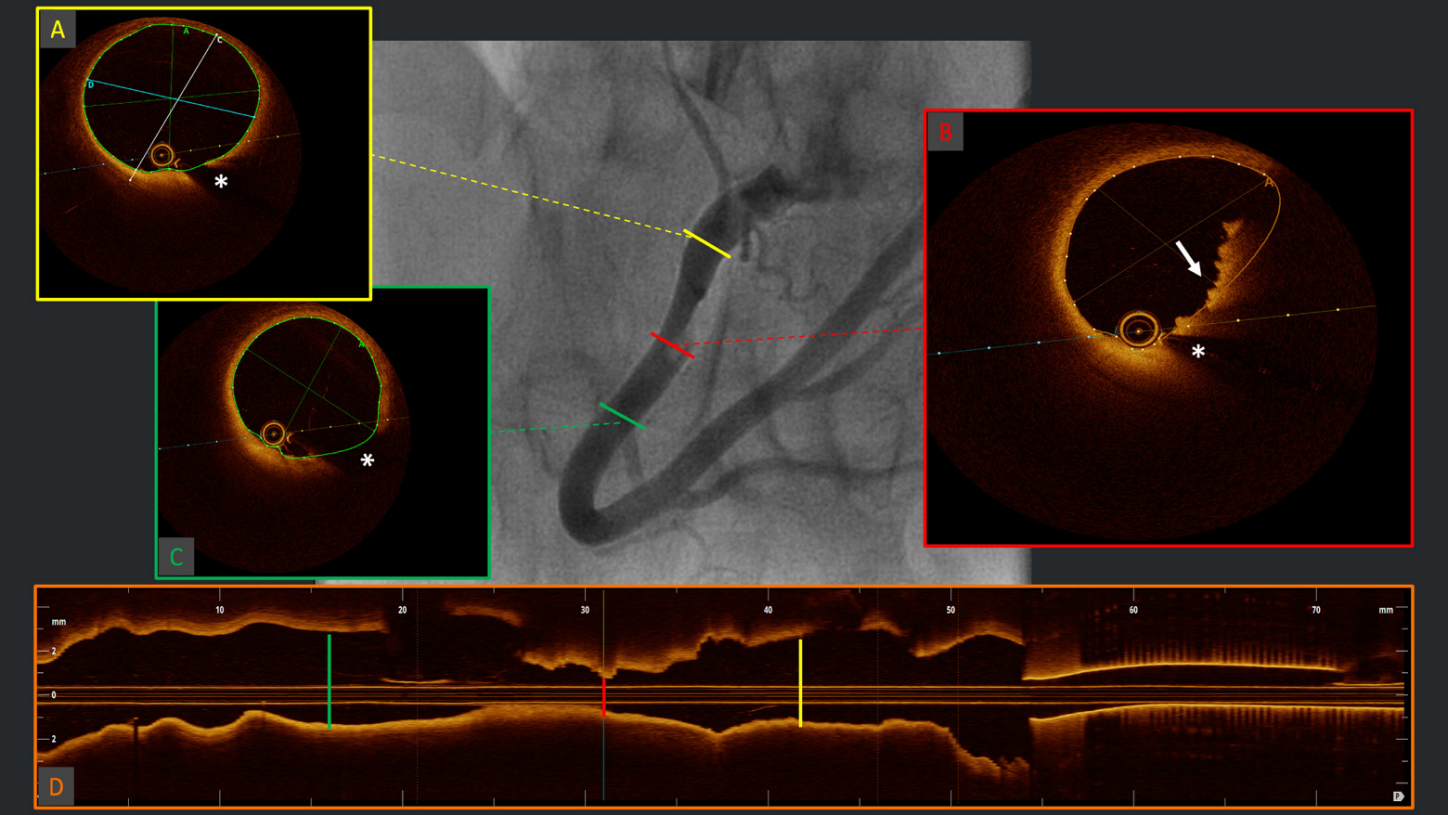
**Diagnosis of plaque erosion**. Example of angiographic acquisition of right coronary artery with suspected thrombus (“haziness”) in the context of acute coronary syndrome. The respective OCT cross-sectional frames (A–C) and longitudinal reconstruction (D), evidencing (B) red thrombus with typical protrusions in the lumen with high back-scattering and high signal attenuation (white arrow) and healthy vessel walls proximally (A) and distally (C) to the culprit segment. OCT, optical coherence tomography. *Wire artifact.

OCT can provide unique insights into the mechanisms underlying acute coronary syndromes (ACS) by accurately assessing vessel and lumen geometry and identifying the hallmarks of culprit lesions in ST- or non-ST elevation myocardial infarction, such as plaque disruption, intraluminal thrombus, and spotty calcifications [53]. Furthermore, OCT enables the differential diagnosis between plaque rupture and plaque erosion (an example of this is shown in Fig. 2). These two entities, despite their similar presentations, have distinct pathogeneses and treatments. In plaque rupture, luminal thrombosis is triggered by the contact of flowing blood with the highly thrombogenic necrotic core and with tissue factor, which is primarily derived from macrophages that are more prevalent in the case of fibrous cap disruption. In contrast, in the case of plaque erosion, local flow perturbations accompanied by upregulation of Toll-like receptor 2 and subsequent activation of platelets and endothelial denudation lead to occlusive thrombosis [54]. For plaque rupture, performing PCI to seal the prothrombotic cavity is the standard and largely validated approach [55]. In cases of confirmed plaque erosion, a conservative approach, combined with dual antiplatelet therapy, has been shown to be safe [56]. OCT is also critical for defining eruptive calcified nodules, a relatively rare but important cause of ACS with the highest incidence of MACEs, mainly driven by recurrent TLF and ACS at follow-up, with nodule re-protrusion even after stenting [57]. Finally, OCT can be an important tool in the diagnostic work-up of myocardial infarction with non-obstructive coronary arteries (MINOCA): after exclusion of atherosclerotic causes (plaque rupture, plaque erosion, calcified nodules), identification of non-atherosclerotic causes (spontaneous coronary artery dissection, epicardial spasm, thrombus embolization) can be critical for the tailoring of treatment [58], although more data are warranted in this setting.

### 5.4 Assessment of Calcium Burden

**Fig. 3.**
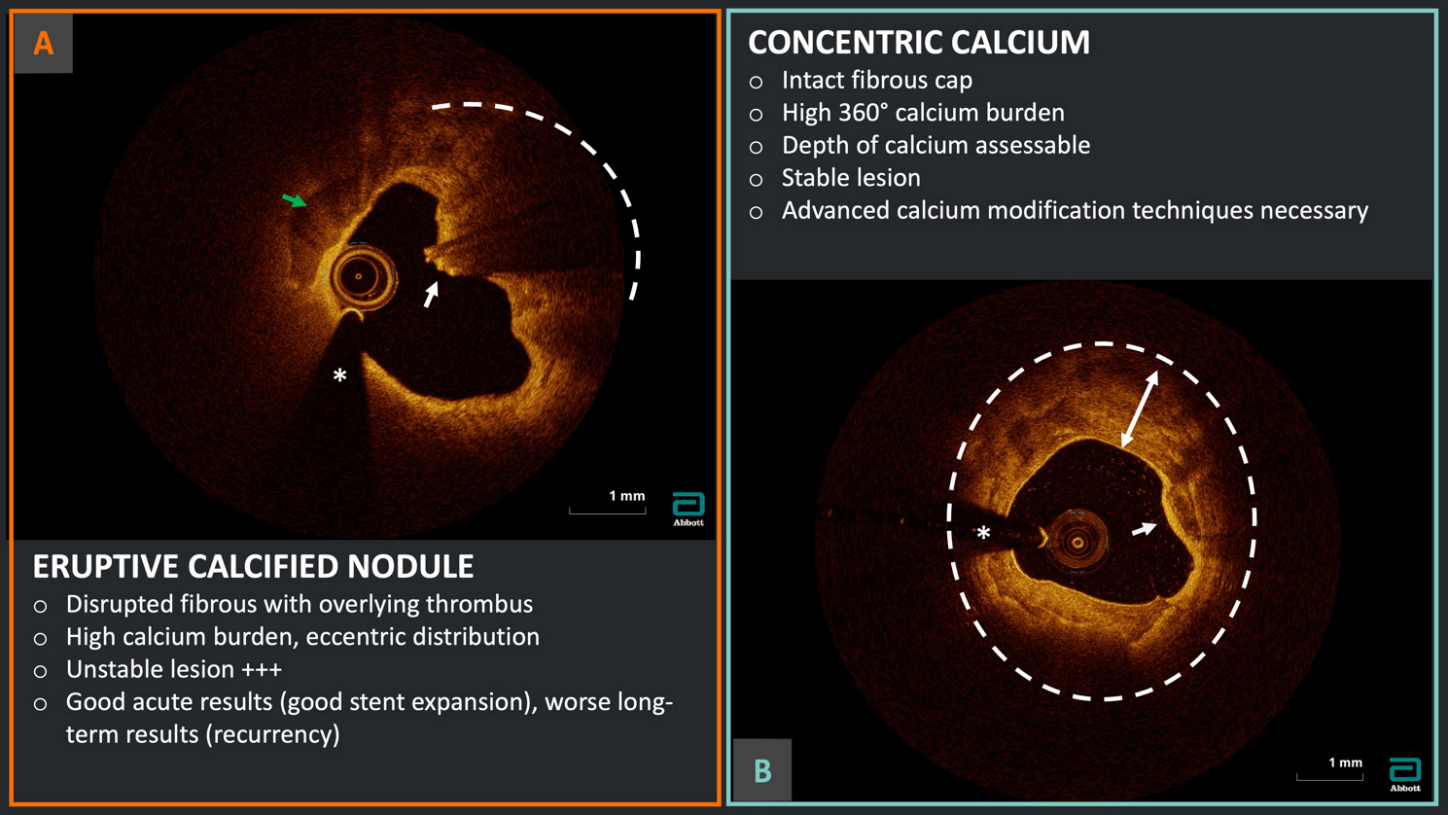
**Calcium burden assessment and characterization**. Example of two different calcium morphologies at OCT analysis: (A) eruptive calcified nodule in the context of acute coronary syndrome with disrupted fibrous cap (white arrow), overlying red thrombus with high attenuation (dashed arc) and evidence of high calcium burden (green arrow); (B) severe 360° calcification (dashed circle) with thick calcium (two-headed arrow) but intact fibrous cap (white arrow) indicating a probable stable lesion. *Wire artifact.

Compared to IVUS, OCT can more precisely characterize calcium morphology, defining not only the arc, length and depth of calcium distribution—fundamental predictors of stent under-expansion [24]—but also allowing critical distinction between eruptive and non-eruptive calcified nodules [59]: the former represents a relatively rare cause of acute coronary syndrome (2–7%) and is characterized by good deformability and good acute result but a high rate of recurrence (in-stent re-protrusion); the latter is characterized by a higher degree of calcification at vessel level, which often requires multimodality plaque modifying technologies (i.e., RotaTripsy, RotaCut, etc., [60, 61]) to achieve good acute results, although show lower rates of TLR at follow-up. Examples of different calcium morphologies at OCT analysis, are shown in Fig. 3.

### 5.5 Assessment of Vulnerable Plaque

The very high resolution of OCT enables the *in vivo* identification of most plaque features associated with a high risk of plaque rupture in post-mortem pathological studies, including thin fibrous caps atheroma (TCFA), large lipid pools, microvessels, microcalcifications, cholesterol crystals, and inflammatory infiltrates. Nevertheless, the presence of OCT-determined high-risk features, including minimum luminal area (MLA) <3.5 mm^2^, TCFA, and lipid-rich plaques with macrophage infiltrates, was found to have a low positive predictive value (19.4%) in evaluating hard clinical outcomes [62]. Evidence from the first applications of hybrid imaging techniques suggests that these techniques could provide a superior insight into plaque vulnerability and more accurately identify high-risk lesions [63]. Near-infrared spectroscopy (NIRS) is a spectroscopic method that measures the absorption spectrum of tissue in the near-infrared range, allowing for a quantitative determination of lipid burden, expressed as the maximum lipid core burden index within a 4 mm coronary section (maxLCBI4mm) [64]. A hybrid NIRS-IVUS catheter was used in the multicenter, natural history PROSPECT II study to assess both the treated culprit and bystander non-culprit lesion(s) in a large cohort of ACS patients (n = 898): high plaque burden (>70%) and MaxLCBI4mm >324 were individuated as the most important independent predictors of patient-level non-culprit lesion-related MACEs at up-to-4-year follow-up [65]. However, the lack of spatial resolution in IVUS did not allow for the evaluation of finer elements, such as cap thickness and neointimal coverage of stent struts, which suffered from relevant loss of signal behind superficial calcium and limited the quantitative analysis to lipidic burden. To address these challenges, a new variety of emerging multimodality intravascular imaging techniques has been proposed and tested *in vivo* using animal studies. Among the many, near-infrared fluorescence (NIRF) and near-infrared autofluorescence (NIRAF), referred to in combination as NIR(A)F, and fluorescence lifetime (FLIM) imaging have shown promising results combined with the high spatial resolution of OCT imaging [64]. Fluorescence imaging uses photoluminescence to measure fluorescence signals that are generated when the endogenous (NIRAF) or exogenous (NIRF) fluorophores in the plaque are excited by light in the NIR range (650 to 900 nm). According to the type of exogenous fluorescent contrast agent administered, NIRF enables the accurate identification of different plaque components, especially focusing on active inflammation and plaque remodeling processes [66]. Conversely, NIRAF captures intrinsic tissue fluorescence mostly related to the accumulation of heme degradation products in the context of plaque hemorrhage, a hallmark of rupturing plaque [67]. A major limitation of current NIR(A)F–OCT is that the measurement of molecule concentration within the plaque is only semiquantitative, and sophisticated algorithms are necessary to compensate for fluorescence signal attenuation, enabling quantitative fluorescence measurement [64]. In contrast to NIRS and NIR(A)F, which use near-infrared light, FLIM uses ultraviolet light to excite fluorophores—excited by ultraviolet (UV) light—each cellular and molecular component within the arterial wall, including macrophages, lipoproteins, collagen, and elastin, emits characteristic fluorescence signal with peculiar decay pattern, defined as florescence lifetime. Thus, FLIM has the potential to accurately explain the structure of important proteins, including collagen and elastin, within the plaque, thereby differentiating between diffuse intimal thickening, pathological intimal thickening, fibrocalcific plaque, and TCFA. However, the main limitation of the current FLIM–OCT catheter is the limited depth of penetration secondary to the use of UV light [68].

The possibility of using next-generation hybrid intravascular imaging modalities to identify high-risk patients who could benefit from plaque-level tailored aggressive medical or interventional treatment could have substantial health and socio-economic implications in the future.

## 6. OCT Imaging Artifacts

The recognition of artifacts is a key element in OCT interpretation and is crucial in pre- and post-PCI evaluations.

These can be schematically categorized into (1) artifacts that originate with light propagation in the catheter, lumen, or vessel wall; (2) artifacts associated with stent struts; (3) artifacts related to catheter location and movement [69].

Sometimes these artifacts are critical in imaging interpretation. The pattern of light dropout, secondary to stent struts, allows differentiation between metallic and bioresorbable platforms: The first ones appear as single leading-edge structures that cast a single expanding broad shadow over the arterial wall, while biodegradable stent strut shadows are normally observed only along the two lateral sides of the strut, leaving a much greater volume of the native tissue structure. Attenuation of light by macrophage OCT accumulations can cause shadows in images that appear falsely as an underlying lipid pool or necrotic core. Thus, a relatively normal artery with a superficial infiltration of macrophages can even appear as a thin-capped fibroatheroma. Other artifacts can be markers of suboptimal quality imaging, due to inadequate blood clearance, irregular signal-rich intraluminal structures can be confounded with thrombotic material, and, from light scattering, which can alter the appearance of metallic stent struts (blooming, merry-go-round, and ghost struts artifacts) [70]. Ghost lines appear as circular features in the OCT frame and are caused by light reflections from at least two interfaces in the catheter. Calibration during catheter preparation is critical to prevent the appearance of these lines. Residual gas bubbles, due to insufficient catheter flushing, can appear as a distinct region of brightness inside the OCT catheter with a corresponding diminished signal in the OCT sector (“dimmed” sector). Finally, the location of the imaging catheter within the vessel lumen (centered vs. off-centered) and the interaction with the vessel anatomy, which can induce uneven rotation and acceleration of the pullback speed, can also determine artifacts. Non-uniform rotational distortion (NURD) is due to a non-constant angular velocity of the mono-fiber optical catheter and appears in OCT images as a blurring or smearing in the lateral or rotational direction. NURD normally occurs due to mechanical rotational resistance in the catheter, resulting from either a tortuous or narrow vessel, a tight hemostatic valve, or a crimped catheter sheath. A sew-up artifact occurs when the combined rotational and longitudinal motions of the catheter result in a discontinuity between successive B-scans. The catheter is oblique to the vessel axis, which places it closer to the artery wall in some longitudinal positions and further in others, resulting in a falsely elliptical lumen. A fold-over artifact is observed when the vessel diameter is too large for the penetration depth of the imaging catheter. Thus, a fold-over artifact is more common in large vessels or in the presence of tortuous and/or calcified anatomies, where delivery of the system in the distal vessel can be challenging [69].

The new iteration of the Abott™ imaging catheter (Dragonfly OpStar™) has been commercialized to enhance pushability, crossability, and trackability. This has been achieved through a redesign of the shaft, guidewire rail, and guidewire exit port, allowing the catheter to navigate more challenging anatomies while preserving imaging quality [71].

## 7. Current Limitations of OCT

The use of OCT in clinical practice is invaluable; however, for accurate interpretation and appropriate clinical application, the optimal quality of image acquisition and knowledge of the technological limitations are crucial.

The light emitted by the OCT catheter is absorbed by blood; thus, the need for additional contrast administration to allow complete blood clearance is among the most impactful and inherent limitations of OCT in everyday clinical activity [70]. Several biologically safe flushing media have already been tested in clinical practice as alternatives to contrast agents to expand the use of OCT in patients at higher risk of contrast-induced nephropathy. Subsequent *in vitro* and *in vivo* studies have shown comparable image quality to that of contrast with heparinized saline or different dilutions of Ringer’s lactate, starch, or dextran, for both qualitative and quantitative analyses [72, 73, 74, 75, 76]. However, a potentially higher rate of insufficiently cleared imaging, secondary to the less efficient blood clearance, especially in the left coronary artery, and a systematic underestimation of both calculated diameters and areas must be considered. Nevertheless, the abovementioned drawbacks can be potentially overcome with some practical measures: (1) an increase in flushing flow rate and injection duration can compensate for a reduction in fluid viscosity since a lower viscosity solution may have a longer fluid transition zone; notably, once the successful displacement of blood has been achieved, it can be relatively easily maintained; (2) the use of a guide extension catheter allows a complete ostial engagement with minimal loss of flushing solution outside the target vessel and improved blood clearance; (3) the use of correction factors, which consider the varying refractory indices, has been shown to allow great concordance between measurements acquired using contrast or alternative flushing solutions. Another relevant limitation of the currently available OCT technology is the limited depth of field, determined by the necessity of a high-frequency near-infrared light source, and is defined by tissue absorption characteristics and the refractive index of the interface between the catheter and the vessel wall. Current intravascular OCT systems use a central wavelength of approximately 1300 nm; thus, the light can penetrate through most constituents in the arterial wall to a depth of no more than 2–3 mm [16, 77]. Therefore, in larger vessels, the loss of signal, with a higher chance of fold-over artifact, hampers imaging interpretation and clinical utility in large proximal vessels, such as the left main stem. Aorto-ostial lesions are even more challenging: the combination of large vessels and less efficient blood clearance, secondary to the disengagement of the guiding catheter necessary for ostium evaluation, requires the use of guide extension catheters to allow selective contrast delivery and reasonable image quality, although it should be taken into consideration that the material of the distal guiding catheter is mostly not near-infrared transparent and a short (1–2 mm) blind spot at the level of the radiopaque marker of the distal tip is present in every commercially available device [78]. Nonetheless, anecdotal reports of successful guide extension facilitated OCT-guided PCI of aorto-ostial lesions have been published, particularly via the soft tip and the relatively wide-gap helical coil reinforcement of the Telescope® (Medtronic) guide extension catheter [79]. However, in these settings, IVUS remains the preferred option due to its deeper tissue penetration and improved visualization of the coronary ostium [18].

The opposite problem is that very tight stenoses can determine suboptimal image acquisition due to insufficient blood clearance of the distal vessel, thereby affecting the interpretation of coronary wall anatomy and introducing significant changes to the stent strut appearance (merry-go-round, blooming, and ghost strut artifacts). Predilation is often required, inducing iatrogenic modifications of the vessel wall anatomy such as longitudinal and radial plaque re-distribution with new dissection planes. The most widely used commercial OCT imaging catheter, the Dragonfly Opstar/Optis™ by Abbott™, is characterized by a shaft distal diameter of 2.7 F (0.9 mm) [13]. Therefore, distal flushing is almost impossible in cases of stenosis with a minimal lumen diameter of ≤1 mm, and predilation is mandatory, which can alter plaque composition and potentially have a detrimental impact on preprocedural guidance and decision-making. Recently, an alternative technique, the D-PUSH technique, has been proposed to overcome this issue through (1) positioning the intravascular imaging catheter proximal to the lesion; (2) starting the flushing media infusion; (3) after two seconds of injection, the catheter is advanced distal to the lesion; (4) starting automatic pullback from distal to proximal is started [80].

Understanding these limitations of OCT is crucial for selecting the appropriate patients and understanding how to maximize the benefits of IVI in clinical practice.

## 8. Next-Generation Devices

In recent years, the competition among companies producing IVI systems has been significant, driving continuous improvements and advancements in the technology. Comparatively, the competition in the OCT field has been mostly limited to the research sector, especially in the Western world. However, a tide of next-generation devices is expected to reach the clinical market in the future, marking a crucial shift in paradigm for IVI.

In the landscape of IVI technologies, Gentuity (Sudbury, USA) has recently received Food and Drug Administration (FDA) clearance for its High-Frequency™ OCT (HF-OCT™) system, designed to acquire images at a higher speed using a reduced-size imaging catheter. This proprietary Vis-Rx Micro Imaging Catheter is a rapid-exchange catheter characterized by a miniaturized lens within a sheath with a maximal outer diameter of 1.8 F and a cross-sectional area of 0.28 mm^2^ (~50% reduction compared to current technologies). The system allows for vessels as small as 1.3 mm in diameter to be scanned; however, thanks to the high-speed near-infrared laser (A-scan line rate of 200 kHz vs. 90 kHz standard) with extended scan range, the system simultaneously supports the imaging of vessels up to 6–7 mm in diameter with an axial resolution of ~10 µm. Moreover, the fast-rotating optical fiber permits the interrogation of a 100 mm-long segment (~33% longer than other currently available systems) in approximately 1 second [81]. The fast pullback could potentially support a routine approach to CAD with OCT, allowing the use of manual injection with a simple 10 cc syringe and the administration of intracoronary saline (since the necessary time of blood clearance is shortened). The first in-human study showed that HF–OCT can efficaciously visualize segments of severely stenosed coronary arteries before any mechanical manipulation, without compromising image quality. In particular, in a series of 31 vessels analyzed before PCI (without predilation), the average clear image length (defined as % of segments with at least 270° of the vessel distinctly visible) was 87 ± 13% with no difference across other degrees of stenosis (smallest calculated MLA 0.35 mm^2^) [82]. Additionally, pilot *ex vivo* and *in vitro* experiences using HF–OCT for neurovascular treatment demonstrated an excellent performance in terms of imaging clarity despite the high degrees of tortuosity encountered (with no observed NURD artifacts) [81]. The technology is promising, but more clinical data are necessary to confirm the improved performance of this technology.

The next important new contender in the OCT field is represented by the HyperVue™ imaging system by SpectraWave (Bedford, USA). The combination of deepOCT™ and NIRS could potentially lead to a breakthrough. The first in-human experience demonstrated how the greater depth field granted by DeepOCT™ can allow the delineation of the entire coronary vessel, including the external elastic lamina, even in the presence of thick calcium and lipid cores (e.g., calcified nodules), which is critical for optimal PCI guidance [83]. Moreover, in this pilot study, all images were acquired through a saline flush, thanks to the rapid pullback (100 mm at 60 or 120 mm/second speed). Furthermore, the automatic NIRS co-registration for lipid detection could introduce a crucial stratification element in vulnerable plaque detection, solving the primary issue of low resolution and suboptimal plaque characterization encountered with previous IVUS–NIRS devices, and potentially leading to a significant leap in CAD management [84]. Finally, the imaging system is characterized by a lower profile (2.5 F) rapid-exchange catheter with a shorter lens-to-tip length (14 mm vs. 23 mm for current devices), allowing safer imaging in distal, smaller vessels (FDA approval for imaging of vessels from 2.0 to 5.2 mm) [83].

Ultimately, the hybrid IVUS–OCT system (Novasight™ Hybrid) from Conavi Medical (Toronto, Canada) has been introduced as the “final answer” to the enduring conundrum faced by interventional cardiologists who must choose between IVUS and OCT. Thus, several hybrid catheters combining the two modalities have been tested in *ex vivo* and pre-clinical settings. However, the previous devices were characterized by a design either with a side-by-side OCT prism and US transducer, resulting in an excessively rigid and bulky catheter, or a hybrid probe with a 2 mm longitudinal offset, leading to suboptimal image co-registration [85]. In the Novasight™ Hybrid system, the 40 MHz ultrasound transducer is located at the tip of the catheter, where an embedded OCT fiber optic is also situated, providing co-linear acoustic and optical beams and imaging the same cross-section simultaneously [86]. The imaging catheter presents a 1.7 F entry profile that reaches 2.8 F at the level of the imaging window, which is positioned 11.5 mm from the tip. The system allows a co-registered IVUS–OCT pullback with a customizable length of up to 100 mm at 10 or 25 mm/second [87].

**Fig. 4.**
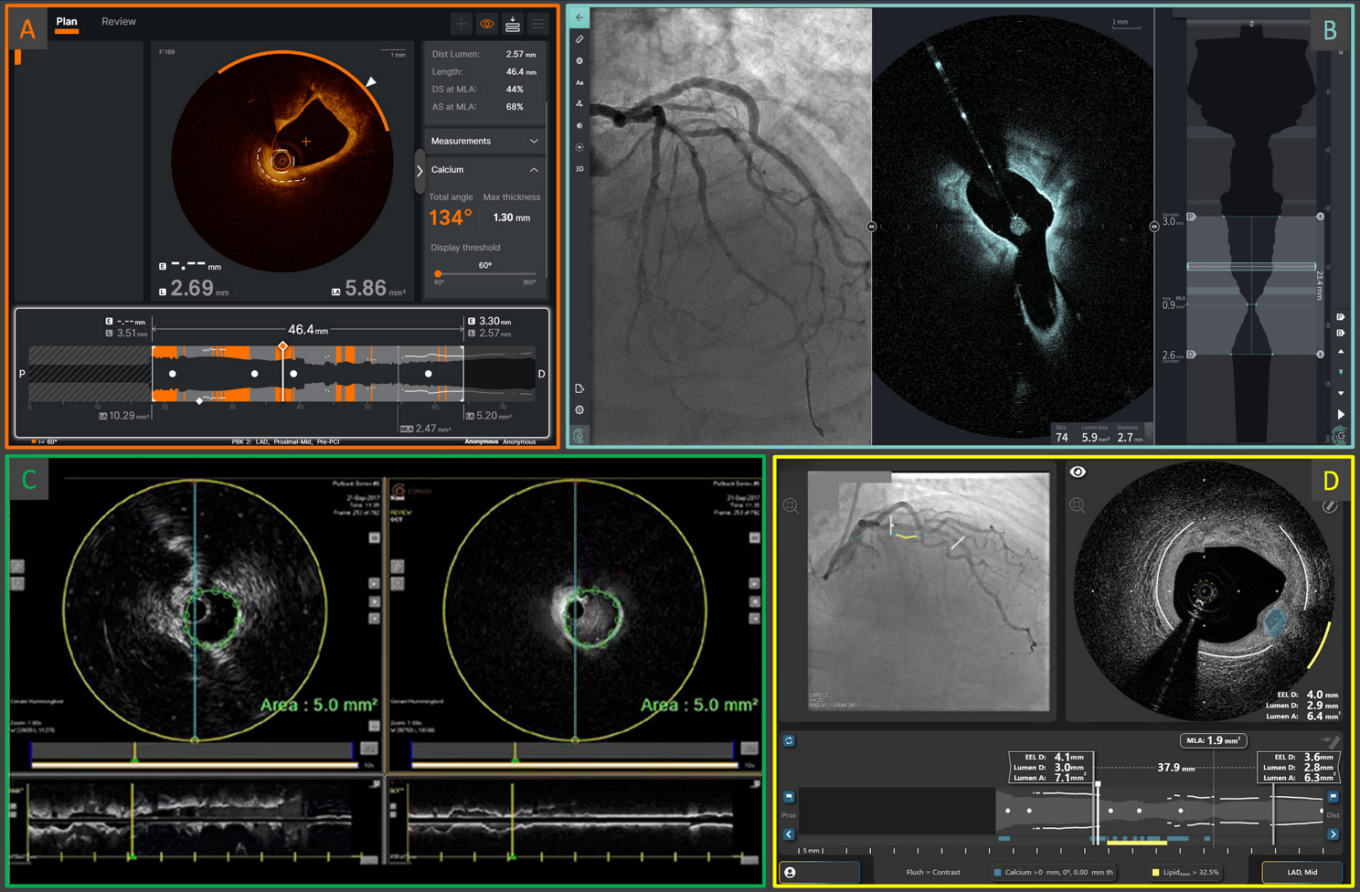
**Examples of the screen interfaces of the current Abbott Ultreon™ 2.0 (A) and the next-generation OCT systems: Gentuity High-Frequency™ OCT (B), Conavi Novasight™ Hybrid (C), and Spectrawave HyperVue™ (D)**. The integration of AI-powered automatic qualitative and quantitative plaque assessments, and/or the implementation of multimodality imaging, are cornerstone elements of next-generation devices. AI, artificial intelligence; OCT, optical coherence tomography. (C) is adapted from Ono M *et al*. [86] “Advances in IVUS/OCT and Future Clinical Perspective of Novel Hybrid Catheter System in Coronary Imaging”. Front Cardiovasc Med. 2020 Jul 31; 7: 119. doi: 10.3389/fcvm.2020.00119. (D) is adapted from Ali ZA *et al*. [83] “First-in-Human Experience With a Novel Multimodality DeepOCT-NIRS Intracoronary Imaging System”. J Soc Cardiovasc Angiogr Interv. 2024 Mar 5; 3(4): 101344. doi: 10.1016/j.jscai.2024.101344. PMID: 39130176; PMCID: PMC11308831.

All the aforementioned systems represent significant advancements in the evolution of intravascular coronary imaging. An overview of the available main technical features and characteristics of these next-generation devices is presented in Table 3, along with examples of the respective screen interfaces in Fig. 4 (Ref. [83, 86]). Challenges such as high costs and the need for specialized training must be further addressed in the future to fully capture the potential of OCT and provide a comprehensive management of both primary and secondary prevention of CAD.

**Table 3.** **Technical comparison between current- and next-generation OCT devices**.

	ABBOTT	GENTUITY	SPECTRA WAVE	CONAVI
	Optis	HighFrequency-OCT	HyperVue	Novasight Hybrid system
Imaging catheter characteristics	DragonFly Opstar® Catheter	Vis-Rx® Micro-Imaging Catheter	Starlight® Imaging Catheter	Novasight Hybrid® Catheter
	Distal tip diameter: 2.7 F	Distal tip diameter: 1.8 F	Distal tip diameter: 2.5 F	Distal tip diameter: 1.7 F (imaging head 2.8 F)
	Lens-to-tip distance: 23 mm	Lens-to-tip distance: 26 mm	Lens-to-tip distance: 14 mm	Lens-to-tip distance: 11.5 mm
Pullback length	54 mm (high resolution)	100 mm	100 mm	Customizable, up to 100 mm
	75 mm (survey)			
Pullback speed	18–36 mm/s	100 mm/s	60–120 mm/s	IVUS-only: 5–1–0.5 mm/s
				IVUS–OCT: 10–25 mm/s
Vessel size range*	2.0–3.5 mm	1.3–6 mm	2.0–5.2 mm	IVUS (40 MHz): up to 6 mm
				OCT: 2.0–3.5 mm
Specialty	AI-automated quantitative analysis (Ultreon ® 2.0)	Low-profile tip, deep tissue penetration, AI-automated quantitative analysis	NIRS, AI-automated quantitative analysis	IVUS–OCT co-planar registration

AI, artificial intelligence; F, french gauge; IVUS, intravascular ultrasound; OCT, optical coherence tomography; NIRS, near-infrared spectroscopy. * FDA-approved adequate vessel size for imaging.

## 9. Artificial Intelligence and the Integration of Imaging and Physiology

As medical technology constantly evolves, new tools emerge to face the challenges in the catheterization laboratory. Interventional cardiologists should be familiar with OCT technology and imaging interpretation; however, AI is becoming increasingly available to enhance diagnostic and/or therapeutic workflows and further optimize clinical management. The advent of AI plays a crucial role in the IVI field, enabling it to overcome challenges in the imaging interpretation process through innovative techniques in image processing, feature extraction, plaque identification, and automated quantitative analysis. For instance, the new version of the Abbott image review software (Ultreon™ 2.0) features AI-powered automated detection of calcium and external elastic lamina (EEL), reducing the need for physicians to identify these structures manually. This system also provides the possibility of combined angiographic and OCT co-registration, further reducing the need for operator interpretation. The benefit of an AI-assisted image interpretation supplied by the Ultreon™ 2.0 software was shown in the study by Cioffi *et al*. [88] where 18 operators enrolled and, after categorization according to experience with OCT analysis, were tasked with reviewing OCT images from both Ultreon™ 2.0 platform and the previous AptiVue™ software, while their eye movements were recorded. The results showed that, in both experienced and inexperienced operators, the AI-powered software improved total task time and streamlined the interpretation process.

**Fig. 5.**
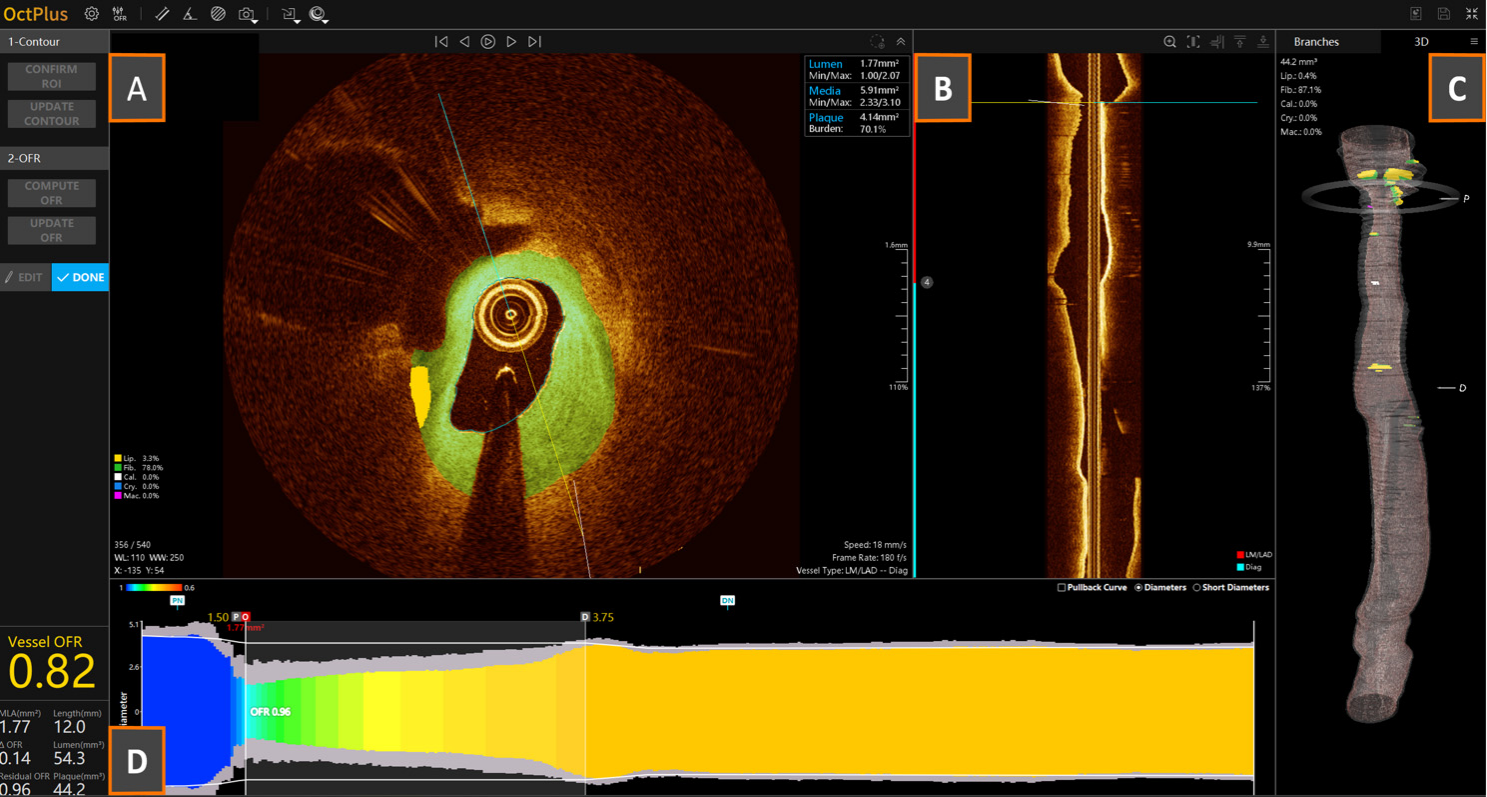
**Example of an OCT-derived optical flow ratio (OFR) analysis**. (A) Cross-sectional OCT frame with AI-based automatic tissue characterization (green = fibrotic tissue; yellow = lipidic tissue); (B) longitudinal OCT reconstruction for vessel navigation and individuation of reference anatomical structures; (C) automatic 3D vessel reconstruction; (D) color-scale-based longitudinal physiological pullback pressure gradient derived by OCT.

Additionally, AI might be even more useful in standardizing and automating anatomical characterization. Chu *et al*. [89] have presented an advanced AI framework for automatic plaque assessment based on a deep-learning convolutional neural network with unique features (e.g., the integration of spatial information from contiguous cross-sections) and trained on a large series of real-world OCT pullbacks. In the validation study, the AI model demonstrated a fast computational speed and excellent performance, with diagnostic accuracies of 97.6%, 90.5%, and 88.5% for fibrous, lipidic, and calcified plaques, respectively. Moreover, the model performed very well in the external validation analysis, with a strong correlation with the core laboratory analysis (R^2^ = 0.98; *p* < 0.001). However, some subsets of lesions were excluded (e.g., plaques with signs of instability, such as thrombus, plaque rupture, dissection, or hematoma), and post-PCI pullbacks were not included in the analysis. The machine-learning model was finally implemented into the OctPlus™ platform (Pulse Medical, Shanghai, China), which allows simultaneous two-dimensional qualitative and quantitative cross-sectional analyses, 3D reconstruction, and automatic OCT-derived physiological assessment (OFR, optical flow ratio). The AI-augmented integration of anatomy and physiology represents the next step in the IVI field in achieving a state-of-the-art patient- and lesion-tailored treatment. The most important step in this image-based FFR approach is to create an accurate 3D geometrical model from imaging data. Hence, OCT, with its superior *in vivo* image resolution, could be a crucial element in the development of this methodology. OFR has already been tested using both pre- and post-PCI settings, demonstrating good to excellent diagnostic accuracy compared to invasive FFR with preserved low inter- and intra-observer variability [90, 91, 92]. A very good vessel-level diagnostic concordance between OFR and FFR was confirmed in the individual patient data meta-analysis by Hu *et al*. [93] (pre-PCI: 91%; post-PCI: 87%; overall: 90%). However, prognostic data remain limited. Despite the current limitations, according to the initial data results, OCT imaging systems with faster pullback speeds and longer pullback lengths might further reduce the discrepancy between OFR and FFR. An example of an OFR analysis is shown in Fig. 5.

Concomitantly, a novel OCT-derived physiology index by Abbott, namely virtual flow reserve (VFR), based on a lumped model of flow-limiting resistors representing the flow losses due to lesions of the main vessel and side branches, and integrating the microvascular resistances modelled for each vessel, has been presented and tested in the multicenter, single-arm prospective FUSION (Validation of OCT-Based Functional Diagnosis of Coronary Stenosis) study—the current largest prospective study with OCT-derived physiology implementation [94]. In this study, a cohort of 266 vessels in 312 patients with stable or unstable CAD and intermediate angiographic stenosis, with an indication for physiological assessment, was investigated through both invasive FFR and OCT + VFR analyses. The authors reported high accuracy for VFR and good correlation with FFR, despite a significant limitation due to the restricted length of the OCT pullback. However, the authors emphasized that 25% of patients changed their treatment allocation based on the physiological findings, and a different device size was used in more than half of the population compared to the original treatment plan, which was based solely on angiographic assessment. Thus, in the future, the synergistic integration of physiology and imaging could represent a game changer in the therapeutic workflow of CAD.

## 10. Future Perspectives of OCT: Conclusions

The future of IVI and OCT is promising. Indeed, evidence of the positive impact of IVI guidance has increased not only for vessel-oriented outcomes but also on clinically relevant hard endpoints, leading to upgraded recommendations in international guidelines. Understanding and addressing the limitations of current technologies are critical steps in enhancing the accuracy of image acquisition and analysis, as well as optimizing the use of OCT in a clinical context. The forthcoming next-generation devices have the potential to provide breakthrough insights into atherosclerotic pathobiology and further improve these outcomes, thanks to the evolution of hybrid multimodality imaging catheters. Improved deliverability, increased penetration field of vision, and the possibility of using saline instead of contrast as a standard flushing agent will further broaden the clinical utility of OCT. Concomitantly, a deeper integration of AI will allow automatic plaque composition and integrated anatomical and physiological assessment, simplifying imaging interpretation and improving the ad-hoc decision-making process, even in high-case-load catheterization laboratories. Further efforts by national and international professional societies, in collaboration with local hospital administrations, are necessary to promote education, dispel misconceptions, and increase the adoption of IVI, thereby improving patient outcomes and potentially achieving a long-term beneficial social health impact.

## References

[1] Stark B, Johnson C, Roth GA (2024). Global prevalence of coronary artery disease: an update from the global burden of disease study. *Journal of the American College of Cardiology*.

[2] Tajeu GS, Ruiz-Negrón N, Moran AE, Zhang Z, Kolm P, Weintraub WS (2024). Cost of Cardiovascular Disease Event and Cardiovascular Disease Treatment-Related Complication Hospitalizations in the United States. *Circulation. Cardiovascular Quality and Outcomes*.

[3] Barton M, Grüntzig J, Husmann M, Rösch J (2014). Balloon Angioplasty - The Legacy of Andreas Grüntzig, M.D. *Frontiers in Cardiovascular Medicine*.

[4] Mintz GS, Matsumura M, Ali Z, Maehara A (2022). Clinical Utility of Intravascular Imaging: Past, Present, and Future. *JACC. Cardiovascular Imaging*.

[5] Gurav A, Revaiah PC, Tsai TY, Miyashita K, Tobe A, Oshima A (2024). Coronary angiography: a review of the state of the art and the evolution of angiography in cardio therapeutics. *Frontiers in Cardiovascular Medicine*.

[6] Escaned J, Lombardi M, Götberg M, Amabile N, Banning A, Barbato E (2025). Factors Contributing to Low Utilization of Intracoronary Imaging in Clinical Practice: A White Paper. *Journal of the Society for Cardiovascular Angiography & Interventions*.

[7] Kuno T, Numasawa Y, Sawano M, Abe T, Ueda I, Kodaira M (2019). Real-world use of intravascular ultrasound in Japan: a report from contemporary multicenter PCI registry. *Heart and Vessels*.

[8] Koskinas KC, Nakamura M, Räber L, Colleran R, Kadota K, Capodanno D (2018). Current use of intracoronary imaging in interventional practice – Results of a European Association of Percutaneous Cardiovascular Interventions (EAPCI) and Japanese Association of Cardiovascular Interventions and Therapeutics (CVIT) Clinical Practice Survey. *EuroIntervention*.

[9] Malik AO, Saxon JT, Spertus JA, Salisbury A, Grantham JA, Kennedy K (2023). Hospital-Level Variability in Use of Intracoronary Imaging for Percutaneous Coronary Intervention in the United States. *Journal of the Society for Cardiovascular Angiography & Interventions*.

[10] Maresca D, Adams S, Maresca B, van der Steen AFW (2014). Mapping intravascular ultrasound controversies in interventional cardiology practice. *PloS One*.

[11] Sharp ASP, Kinnaird T, Curzen N, Ayyub R, Alfonso JE, Mamas MA (2024). Cost-effectiveness of intravascular ultrasound-guided percutaneous intervention in patients with acute coronary syndromes: a UK perspective. *European Heart Journal. Quality of Care & Clinical Outcomes*.

[12] Takura T, Komuro I, Ono M (2023). Trends in the cost-effectiveness level of percutaneous coronary intervention: Macro socioeconomic analysis and health technology assessment. *Journal of Cardiology*.

[13] Hong D, Lee J, Lee H, Cho J, Guallar E, Choi KH (2024). Cost-Effectiveness of Intravascular Imaging-Guided Complex PCI: Prespecified Analysis of RENOVATE-COMPLEX-PCI Trial. *Circulation. Cardiovascular Quality and Outcomes*.

[14] Miller MK, Clark JD, Jehle A (2015). Cognitive Dissonance Theory (Festinger). *The Blackwell Encyclopedia of Sociology*.

[15] Yonetsu T, Jang IK (2023). Cardiac Optical Coherence Tomography: History, Current Status, and Perspective. *JACC. Asia*.

[16] Fujimoto JG, Schmitt J, Swanson E, Aguirre AD, Jang I-K, Jang I-K (2020). The Development of Optical Coherence Tomography. *Cardiovascular OCT Imaging*.

[17] Araki M, Park SJ, Dauerman HL, Uemura S, Kim JS, Di Mario C (2024). Optical coherence tomography in coronary atherosclerosis assessment and intervention [published correction in Nature Reviews Cardiology. 2024; 21: 348. https://doi.org/10.1038/s41569-023-00982-z]. *Nature Reviews Cardiology*.

[18] Maehara A, Matsumura M, Ali ZA, Mintz GS, Stone GW (2017). IVUS-Guided Versus OCT-Guided Coronary Stent Implantation: A Critical Appraisal. *JACC. Cardiovascular Imaging*.

[19] Adlam D, Tweet MS, Gulati R, Kotecha D, Rao P, Moss AJ (2021). Spontaneous Coronary Artery Dissection: Pitfalls of Angiographic Diagnosis and an Approach to Ambiguous Cases. *JACC. Cardiovascular Interventions*.

[20] Xenogiannis I, Pavlidis AN, Kaier TE, Rigopoulos AG, Karamasis GV, Triantafyllis AS (2023). The role of intravascular imaging in chronic total occlusion percutaneous coronary intervention. *Frontiers in Cardiovascular Medicine*.

[21] Tanaka K, Okamura A, Yoshikawa R, Tsuchikane E, Ishikawa M, Suzuki S (2024). Tip Detection-Antegrade Dissection and Re-Entry With New Puncture Wire in CTO Intervention. *JACC Asia*.

[22] Shabbir A, Ali Z, Colletti G, Dudek D, Garbo R, Hellig F (2025). Ultra-Low-Contrast PCI: A Structured Approach to Reducing Dependence on Contrast Vessel Opacification in PCI. *JACC. Cardiovascular Interventions*.

[23] Vergallo R, Jang I-K, Jang I-K (2020). Detection of Vulnerable Plaque. *Cardiovascular OCT Imaging*.

[24] Fujino A, Mintz GS, Matsumura M, Lee T, Kim SY, Hoshino M (2018). A new optical coherence tomography-based calcium scoring system to predict stent underexpansion. *EuroIntervention: Journal of EuroPCR in Collaboration with the Working Group on Interventional Cardiology of the European Society of Cardiology*.

[25] Andreasen LN, Balleby IR, Barkholt TØ, Hebsgaard L, Terkelsen CJ, Holck EN (2023). Early healing after treatment of coronary lesions by thin strut everolimus, or thicker strut biolimus eluting bioabsorbable polymer stents: The SORT-OUT VIII OCT study. *Catheterization and Cardiovascular Interventions: Official Journal of the Society for Cardiac Angiography & Interventions*.

[26] Cioffi GM, Lamelas P, Shenouda M, Halperin J, Goffredo F, McGrath BP (2025). OCT-based diagnosis, management, and predictors of recurrent stent failure: a cohort study. *Frontiers in Cardiovascular Medicine*.

[27] Jones DA, Rathod KS, Koganti S, Hamshere S, Astroulakis Z, Lim P (2018). Angiography Alone Versus Angiography Plus Optical Coherence Tomography to Guide Percutaneous Coronary Intervention: Outcomes From the Pan-London PCI Cohort. *JACC: Cardiovascular Interventions*.

[28] Kim N, Lee JH, Jang SY, Bae MH, Yang DH, Park HS (2020). Intravascular modality-guided versus angiography-guided percutaneous coronary intervention in acute myocardial infarction. *Catheterization and Cardiovascular Interventions: Official Journal of the Society for Cardiac Angiography & Interventions*.

[29] Prati F, Di Vito L, Biondi-Zoccai G, Occhipinti M, La Manna A, Tamburino C (2012). Angiography alone versus angiography plus optical coherence tomography to guide decision-making during percutaneous coronary intervention: the Centro per la Lotta contro l’Infarto-Optimisation of Percutaneous Coronary Intervention (CLI-OPCI) study. *EuroIntervention: Journal of EuroPCR in Collaboration with the Working Group on Interventional Cardiology of the European Society of Cardiology*.

[30] Lee JM, Choi KH, Song YB, Lee JY, Lee SJ, Lee SY (2023). Intravascular Imaging-Guided or Angiography-Guided Complex PCI. *The New England Journal of Medicine*.

[31] Holm NR, Andreasen LN, Neghabat O, Laanmets P, Kumsars I, Bennett J (2023). OCT or Angiography Guidance for PCI in Complex Bifurcation Lesions. *The New England Journal of Medicine*.

[32] Ali ZA, Landmesser U, Maehara A, Matsumura M, Shlofmitz RA, Guagliumi G (2023). Optical Coherence Tomography-Guided versus Angiography-Guided PCI. *The New England Journal of Medicine*.

[33] Hong SJ, Lee SJ, Lee SH, Lee JY, Cho DK, Kim JW (2024). Optical coherence tomography-guided versus angiography-guided percutaneous coronary intervention for patients with complex lesions (OCCUPI): an investigator-initiated, multicentre, randomised, open-label, superiority trial in South Korea. *Lancet (London, England)*.

[34] Khan SU, Agarwal S, Arshad HB, Akbar UA, Mamas MA, Arora S (2023). Intravascular imaging guided versus coronary angiography guided percutaneous coronary intervention: systematic review and meta-analysis. *BMJ (Clinical Research Ed.)*.

[35] Kuno T, Kiyohara Y, Maehara A, Ueyama HA, Kampaktsis PN, Takagi H (2023). Comparison of Intravascular Imaging, Functional, or Angiographically Guided Coronary Intervention. *Journal of the American College of Cardiology*.

[36] Giacoppo D, Laudani C, Occhipinti G, Spagnolo M, Greco A, Rochira C (2024). Coronary Angiography, Intravascular Ultrasound, and Optical Coherence Tomography for Guiding of Percutaneous Coronary Intervention: A Systematic Review and Network Meta-Analysis. *Circulation*.

[37] Stone GW, Christiansen EH, Ali ZA, Andreasen LN, Maehara A, Ahmad Y (2024). Intravascular imaging-guided coronary drug-eluting stent implantation: an updated network meta-analysis. *Lancet (London, England)*.

[38] Kubo T, Shinke T, Okamura T, Hibi K, Nakazawa G, Morino Y (2017). Optical frequency domain imaging vs. intravascular ultrasound in percutaneous coronary intervention (OPINION trial): one-year angiographic and clinical results. *European Heart Journal*.

[39] Ali ZA, Karimi Galougahi K, Maehara A, Shlofmitz RA, Fabbiocchi F, Guagliumi G (2021). Outcomes of optical coherence tomography compared with intravascular ultrasound and with angiography to guide coronary stent implantation: one-year results from the ILUMIEN III: OPTIMIZE PCI trial. *EuroIntervention: Journal of EuroPCR in Collaboration with the Working Group on Interventional Cardiology of the European Society of Cardiology*.

[40] Kang DY, Ahn JM, Yun SC, Hur SH, Cho YK, Lee CH (2023). Optical Coherence Tomography-Guided or Intravascular Ultrasound-Guided Percutaneous Coronary Intervention: The OCTIVUS Randomized Clinical Trial. *Circulation*.

[41] Vrints C, Andreotti F, Koskinas KC, Rossello X, Adamo M, Ainslie J (2024). 2024 ESC Guidelines for the management of chronic coronary syndromes. *European Heart Journal*.

[42] Nogic J, Prosser H, O’Brien J, Thakur U, Soon K, Proimos G (2020). The assessment of intermediate coronary lesions using intracoronary imaging. *Cardiovascular Diagnosis and Therapy*.

[43] Burzotta F, Zito A, Aurigemma C, Romagnoli E, Bianchini F, Bianchini E (2024). Fractional flow reserve or optical coherence tomography for angiographically intermediate coronary stenoses: 5-year outcomes in the FORZA trial. *European Heart Journal*.

[44] Roule V, Rebouh I, Lemaitre A, Bignon M, Ardouin P, Sabatier R (2020). Evaluation of Left Main Coronary Artery Using Optical Frequency Domain Imaging and Its Pitfalls. *Journal of Interventional Cardiology*.

[45] Burzotta F, Dato I, Trani C, Pirozzolo G, De Maria GL, Porto I (2015). Frequency domain optical coherence tomography to assess non-ostial left main coronary artery. *EuroIntervention: Journal of EuroPCR in Collaboration with the Working Group on Interventional Cardiology of the European Society of Cardiology*.

[46] Moussa ID, Mohananey D, Saucedo J, Stone GW, Yeh RW, Kennedy KF (2020). Trends and Outcomes of Restenosis After Coronary Stent Implantation in the United States. *Journal of the American College of Cardiology*.

[47] Erdogan E, Bajaj R, Lansky A, Mathur A, Baumbach A, Bourantas CV (2022). Intravascular Imaging for Guiding In-Stent Restenosis and Stent Thrombosis Therapy. *Journal of the American Heart Association*.

[48] Kang DY, Ahn JM, Yun SC, Hur SH, Cho YK, Lee CH (2024). Guiding Intervention for Complex Coronary Lesions by Optical Coherence Tomography or Intravascular Ultrasound. *Journal of the American College of Cardiology*.

[49] Tada T, Byrne RA, Simunovic I, King LA, Cassese S, Joner M (2013). Risk of stent thrombosis among bare-metal stents, first-generation drug-eluting stents, and second-generation drug-eluting stents: results from a registry of 18,334 patients. *JACC. Cardiovascular Interventions*.

[50] Souteyrand G, Amabile N, Mangin L, Chabin X, Meneveau N, Cayla G (2016). Mechanisms of stent thrombosis analysed by optical coherence tomography: insights from the national PESTO French registry. *European Heart Journal*.

[51] Adriaenssens T, Joner M, Godschalk TC, Malik N, Alfonso F, Xhepa E (2017). Optical Coherence Tomography Findings in Patients With Coronary Stent Thrombosis: A Report of the PRESTIGE Consortium (Prevention of Late Stent Thrombosis by an Interdisciplinary Global European Effort). *Circulation*.

[52] Neumann FJ, Sousa-Uva M, Ahlsson A, Alfonso F, Banning AP, Benedetto U (2019). 2018 ESC/EACTS Guidelines on myocardial revascularization. *EuroIntervention: Journal of EuroPCR in Collaboration with the Working Group on Interventional Cardiology of the European Society of Cardiology*.

[53] Sagar H, George J, Joseph V, Joseph J, Abdullakutty J, Mathew R (2023). Optical Coherence Tomography (OCT) evaluation of culprit lesions in patients with Non-ST Elevation Acute Coronary Syndromes (NSTE-ACS). *European Heart Journal*.

[54] Vergallo R, Jang IK, Crea F (2021). New prediction tools and treatment for ACS patients with plaque erosion. *Atherosclerosis*.

[55] Hougaard M, Hansen HS, Thayssen P, Antonsen L, Jensen LO (2018). Uncovered Culprit Plaque Ruptures in Patients With ST-Segment Elevation Myocardial Infarction Assessed by Optical Coherence Tomography and Intravascular Ultrasound With iMap. *JACC. Cardiovascular Imaging*.

[56] Xing L, Yamamoto E, Sugiyama T, Jia H, Ma L, Hu S (2017). EROSION Study (Effective Anti-Thrombotic Therapy Without Stenting: Intravascular Optical Coherence Tomography-Based Management in Plaque Erosion): A 1-Year Follow-Up Report. *Circulation. Cardiovascular Interventions*.

[57] Kondo S, Mizukami T, Kobayashi N, Wakabayashi K, Mori H, Yamamoto MH (2023). Diagnosis and Prognostic Value of the Underlying Cause of Acute Coronary Syndrome in Optical Coherence Tomography-Guided Emergency Percutaneous Coronary Intervention. *Journal of the American Heart Association*.

[58] Karamasis GV, Xenogiannis I, Varlamos C, Deftereos S, Alexopoulos D (2022). Use of Optical Coherence Tomography in MI with Non-obstructive Coronary Arteries. https://www.icrjournal.com/articles/use-optical-coherence-tomography-mi-non-obstructive-coronary-arteries?language_content_entity=en.

[59] Shin D, Karimi Galougahi K, Spratt JC, Maehara A, Collet C, Barbato E (2024). Calcified Nodule in Percutaneous Coronary Intervention: Therapeutic Challenges. *JACC. Cardiovascular Interventions*.

[60] Gonzálvez-García A, Jiménez-Valero S, Galeote G, Moreno R, López de Sá E, Jurado-Román A (2022). “RotaTripsy”: Combination of Rotational Atherectomy and Intravascular Lithotripsy in Heavily Calcified Coronary Lesions: A Case Series. *Cardiovascular Revascularization Medicine: Including Molecular Interventions*.

[61] Allali A, Toelg R, Abdel-Wahab M, Hemetsberger R, Kastrati A, Mankerious N (2022). Combined rotational atherectomy and cutting balloon angioplasty prior to drug-eluting stent implantation in severely calcified coronary lesions: The PREPARE-CALC-COMBO study. *Catheterization and Cardiovascular Interventions: Official Journal of the Society for Cardiac Angiography & Interventions*.

[62] Prati F, Romagnoli E, Gatto L, La Manna A, Burzotta F, Ozaki Y (2020). Relationship between coronary plaque morphology of the left anterior descending artery and 12 months clinical outcome: the CLIMA study. *European Heart Journal*.

[63] Oemrawsingh RM, Cheng JM, García-García HM, van Geuns RJ, de Boer SPM, Simsek C (2014). Near-infrared spectroscopy predicts cardiovascular outcome in patients with coronary artery disease. *Journal of the American College of Cardiology*.

[64] Li J, Montarello NJ, Hoogendoorn A, Verjans JW, Bursill CA, Peter K (2022). Multimodality Intravascular Imaging of High-Risk Coronary Plaque. *JACC. Cardiovascular Imaging*.

[65] Erlinge D, Maehara A, Ben-Yehuda O, Bøtker HE, Maeng M, Kjøller-Hansen L (2021). Identification of vulnerable plaques and patients by intracoronary near-infrared spectroscopy and ultrasound (PROSPECT II): a prospective natural history study. *Lancet (London, England)*.

[66] Jaffer FA, Libby P, Weissleder R (2009). Optical and multimodality molecular imaging: insights into atherosclerosis. *Arteriosclerosis, Thrombosis, and Vascular Biology*.

[67] Htun NM, Chen YC, Lim B, Schiller T, Maghzal GJ, Huang AL (2017). Near-infrared autofluorescence induced by intraplaque hemorrhage and heme degradation as marker for high-risk atherosclerotic plaques. *Nature Communications*.

[68] Lee MW, Song JW, Kang WJ, Nam HS, Kim TS, Kim S (2018). Comprehensive intravascular imaging of atherosclerotic plaque in vivo using optical coherence tomography and fluorescence lifetime imaging. *Scientific Reports*.

[69] Phipps JE, Hoyt T, Halaney D, Mancuso JJ, Elahi S, Cabe A, Jang I-K (2020). Intravascular OCT Imaging Artifacts. *Cardiovascular OCT Imaging*.

[70] Ali ZA, Karimi Galougahi K, Mintz GS, Maehara A, Shlofmitz RA, Mattesini A (2021). Intracoronary optical coherence tomography: state of the art and future directions. *EuroIntervention: Journal of EuroPCR in Collaboration with the Working Group on Interventional Cardiology of the European Society of Cardiology*.

[71] Miyazaki R, Lee T, Kaneko M, Nagata Y, Nozato T, Ashikaga T (2025). Optical coherence tomography image with the three circles sign caused by the Z-shape phenomenon. *AsiaIntervention*.

[72] Gore AK, Shlofmitz E, Karimi Galougahi K, Petrossian G, Jeremias A, Sosa FA (2020). Prospective Comparison Between Saline and Radiocontrast for Intracoronary Imaging With Optical Coherence Tomography. *JACC. Cardiovascular Imaging*.

[73] Gupta A, Chhikara S, Vijayvergiya R, Seth A, Mahesh NK, Akasaka T (2022). Saline as an Alternative to Radio-Contrast for Optical Coherence Tomography-Guided Percutaneous Coronary Intervention: A Prospective Comparison. *Cardiovascular Revascularization Medicine: Including Molecular Interventions*.

[74] Kimura M, Takeda T, Tsujino Y, Matsumoto Y, Yamaji M, Sakaguchi T (2024). Assessing the efficacy of saline flush in frequency-domain optical coherence tomography for intracoronary imaging. *Heart and Vessels*.

[75] Kang DO, Nam HS, Kim S, Yoo H, Kim JW (2023). Feasibility and safety of non-contrast optical coherence tomography imaging using hydroxyethyl starch in coronary arteries. *Scientific Reports*.

[76] Ozaki Y, Kitabata H, Tsujioka H, Hosokawa S, Kashiwagi M, Ishibashi K (2012). Comparison of contrast media and low-molecular-weight dextran for frequency-domain optical coherence tomography. *Circulation Journal: Official Journal of the Japanese Circulation Society*.

[77] Adriaenssens T, Jang I-K (2020). Basic Interpretation Skills. *Cardiovascular OCT Imaging*.

[78] Dadkhah R, Ungureanu C (2023). Guide extension and optical coherence tomography, a new approach to study aorto-ostial coronary lesions: a case report. *European Heart Journal. Case Reports*.

[79] Kurogi K, Ishii M, Yamamoto N, Yamanaga K, Tsujita K (2021). Optical coherence tomography-guided percutaneous coronary intervention: a review of current clinical applications. *Cardiovascular Intervention and Therapeutics*.

[80] Kobayashi N, Shibata Y, Okazaki H, Shirakabe A, Takano M, Miyauchi Y (2021). A novel technique of low molecular weight dextran infusion followed by catheter push (D-PUSH) for optical coherence tomography. *EuroIntervention: Journal of EuroPCR in Collaboration with the Working Group on Interventional Cardiology of the European Society of Cardiology*.

[81] Ughi GJ, Marosfoi MG, King RM, Caroff J, Peterson LM, Duncan BH (2020). A neurovascular high-frequency optical coherence tomography system enables in situ cerebrovascular volumetric microscopy. *Nature Communications*.

[82] Bezerra HG, Quimby DL, Matar F, Mohanty BD, Bassily E, Ughi GJ (2023). High-Frequency Optical Coherence Tomography (HF-OCT) for Preintervention Coronary Imaging: A First-in-Human Study. *JACC. Cardiovascular Imaging*.

[83] Ali ZA, Dager A, Zúñiga M, Fonseca J, Arana C, Chamié D (2024). First-in-Human Experience With a Novel Multimodality DeepOCT-NIRS Intracoronary Imaging System. *Journal of the Society for Cardiovascular Angiography & Interventions*.

[84] Muller J, Madder R (2020). OCT-NIRS Imaging for Detection of Coronary Plaque Structure and Vulnerability. *Frontiers in Cardiovascular Medicine*.

[85] Terashima M, Kaneda H, Honda Y, Shimura T, Kodama A, Habara M (2021). Current status of hybrid intravascular ultrasound and optical coherence tomography catheter for coronary imaging and percutaneous coronary intervention. *Journal of Cardiology*.

[86] Ono M, Kawashima H, Hara H, Gao C, Wang R, Kogame N (2020). Advances in IVUS/OCT and Future Clinical Perspective of Novel Hybrid Catheter System in Coronary Imaging. *Frontiers in Cardiovascular Medicine*.

[87] Sheth TN, Pinilla-Echeverri N, Mehta SR, Courtney BK (2018). First-in-Human Images of Coronary Atherosclerosis and Coronary Stents Using a Novel Hybrid Intravascular Ultrasound and Optical Coherence Tomographic Catheter. *JACC. Cardiovascular Interventions*.

[88] Cioffi GM, Pinilla-Echeverri N, Sheth T, Sibbald MG (2023). Does artificial intelligence enhance physician interpretation of optical coherence tomography: insights from eye tracking. *Frontiers in Cardiovascular Medicine*.

[89] Chu M, Jia H, Gutiérrez-Chico JL, Maehara A, Ali ZA, Zeng X (2021). Artificial intelligence and optical coherence tomography for the automatic characterisation of human atherosclerotic plaques. *EuroIntervention: Journal of EuroPCR in Collaboration with the Working Group on Interventional Cardiology of the European Society of Cardiology*.

[90] Ding D, Yu W, Tauzin H, De Maria GL, Wu P, Yang F (2021). Optical flow ratio for assessing stenting result and physiological significance of residual disease. *EuroIntervention: Journal of EuroPCR in Collaboration with the Working Group on Interventional Cardiology of the European Society of Cardiology*.

[91] Gutiérrez-Chico JL, Chen Y, Yu W, Ding D, Huang J, Huang P (2020). Diagnostic accuracy and reproducibility of optical flow ratio for functional evaluation of coronary stenosis in a prospective series. *Cardiology Journal*.

[92] Takahashi T, Shin D, Kuno T, Lee JM, Latib A, Fearon WF (2022). Diagnostic performance of fractional flow reserve derived from coronary angiography, intravascular ultrasound, and optical coherence tomography; a meta-analysis. *Journal of Cardiology*.

[93] Hu F, Ding D, Westra J, Li Y, Yu W, Wang Z (2023). Diagnostic accuracy of optical flow ratio: an individual patient-data meta-analysis. *EuroIntervention: Journal of EuroPCR in Collaboration with the Working Group on Interventional Cardiology of the European Society of Cardiology*.

[94] Jeremias A, Maehara A, Matsumura M, Shlofmitz RA, Maksoud A, Akasaka T (2024). Optical Coherence Tomography-Based Functional Stenosis Assessment: FUSION-A Prospective Multicenter Trial. *Circulation. Cardiovascular Interventions*.

